# Supplementary material for: Transcriptional Activation of Chac1 and Other Atf4-Target Genes Induced by Extracellular l-Serine Depletion is negated with Glycine Consumption in Hepa1-6 Hepatocarcinoma Cells
Source: Nutrients. 2020 Oct 2;12(10):3018. doi: 10.3390/nu12103018 (PMC7600170; doi:10.3390/nu12103018)
Supplement: Supplementary file 1 [file nutrients-12-03018-s001.pdf]

nutrients

MDPI

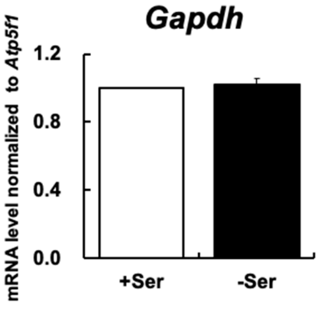


(**a**)

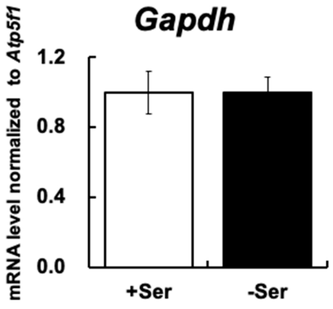


(**b**)

**Figure S1.** The expression levels of Gapdh mRNA were not significantly altered between Ser-supplemented and Ser-depleted in Hep1-6 cells. Hepa1-6 were cultured for 6 h and 24 h under the L-Ser-depleted or -supplemented condition, and *Gapdh* mRNA levels were measured cultured for 6 h (**a**) and for 24h (**b**). *Gapdh* mRNA level were normalized by *Atp5f1* as the internal control. Student's t-test.
